# Optimal timing of endovascular treatment for symptomatic intracranial atherosclerotic stenosis: a real world single center study

**DOI:** 10.3389/fneur.2026.1749046

**Published:** 2026-04-22

**Authors:** Yang Yang, Xiaoya Wang, Jialiang Lu, Ye Li, Lili Zhao, Yating Jian, Tao Li, Meijuan Dang, Ziwei Lu, Fangcun Li, Fan Tang, Qingyu Fan, Ning Bu, Huqing Wang, Ru Zhang, An Wen, Guilian Zhang, Hong Fan, Lei Zhang

**Affiliations:** 1 Department of Neurology, Second Affiliated Hospital of Xi’an Jiaotong University, Xi’an, China; 2 Department of Neurology, Xi’an Daxing Hospital, Xi’an, China; 3 Department of Neurology, Jiangxi Provincial People’s Hospital (The First Affiliated Hospital of Nanchang Medical College), Nanchang, China

**Keywords:** atherosclerosis, intervention, stenosis, stent, time-to-treatment

## Abstract

**Objective:**

To determine the optimal timing of endovascular treatment for acute cerebral infarction with symptomatic intracranial atherosclerotic stenosis (sICAS) in real world practice.

**Methods:**

This retrospective single center study enrolled consecutive sICAS patients undergoing intervention. According to the interval from symptom onset to intervention, patients were divided into early (≤14 days) and delayed (>14 days) intervention groups. Primary outcomes were any stroke or death within 30 days and the 90-day mRS score. Secondary analyses explored factors influencing surgical timing.

**Results:**

Among 211 eligible patients, 174 were analyzed, including 53 patients in the early intervention group and 121 patients in the delayed intervention group. The 30-day stroke or death rates were 11.3 and 8.3%, and unfavorable 90-day outcomes were 18.9 and 10.7% in the early and delayed groups (*p* = 0.521, 0.145). Higher preoperative NIHSS, higher albumin-to-globulin ratio, and higher LDL might be independent factors influencing the doctors’ decision on the timing of the intervention (OR = 0.819, 0.149, 0.394; *p* = 0.027, 0.042, 0.004). Thresholds favoring early intervention were NIHSS ≤ 3, albumin-to-globulin ratio < 1.53, and LDL < 2.85 mmol/L (AUC = 0.664, 0.603, 0.642; *p* = 0.001, 0.030, 0.003). For posterior circulation lesions, early intervention might led to more unfavorable outcomes than delayed intervention (29.2% vs. 8.2%, *p* = 0.033).

**Conclusion:**

In anterior circulation sICAS with minor stroke (NIHSS ≤ 3), high Alberta stroke program early CT score (ASPECTS)/posterior circulation ASPECTS (pcASPECTS) (8–9), and well-controlled LDL, intervention timing may not be restricted, whereas Basilar artery (BA) lesions appear better suited for delayed intervention. Multiple parallel severe stenoses, severe pre-existing global brain injury, and plaque high-signal intensity did not influence on the surgical timing. The conclusions of this study should be validated in future prospective studies.

## 1 Introduction

Intracranial atherosclerotic stenosis (ICAS) is a major cause of stroke worldwide, particularly in East and South Asia, where it is present in 46.6–55.0% of ischemic stroke patients (1–3). The annual stroke risk is approximately 6% for 50–69% stenosis and increases to 19% for 70–99% stenosis (4). Symptomatic ICAS (sICAS), defined as a recent TIA or ischemic stroke attributed to 70–99% atherosclerotic stenosis of a major intracranial artery, carries a 7.2–15.1% risk of recurrent stroke within 1 year despite aggressive medical management (5–7). Therefore, exploring surgical options beyond medical therapy has long been a focus in sICAS treatment.

Endovascular treatment, including stenting and balloon angioplasty alone, has emerged as a promising supplemental treatment for sICAS, yet its efficacy and safety remain debated. Early trials like SAMMPRIS (5) and VISSIT (6), and delayed the CASSISS study (7) its follow-up research spanning up to 7.4 years (8), found no additional benefit of adding angioplasty/stenting to medical therapy alone. It was the BASIS study (9) that established the benefit of balloon angioplasty for sICAS. Furthermore, regarding rescue angioplasty, the ANGEL-REBOOT study found that while the technique did not improve neurological function within 90 days and was associated with increased risks of bleeding and vascular dissection (10), its one-year follow-up results showed a significant reduction in both the recurrence of stroke in the target territory and overall disability levels (11). Differences in outcomes among these studies may be attributed not only to the intervention type (stenting vs. balloon angioplasty) but also to patient selection, operator experience, perioperative management, and notably, the timing of the procedure. A meta-analysis of 12 studies involving 10,107 patients indicated consistent efficacy and safety for both stenting and balloon angioplasty in sICAS (12), suggesting limited impact of the specific technique. Analysis of SAMMPRIS, VISSIT, CASSISS, and BASIS revealed median times from symptom onset to intervention of 7, 9, 35, and 34 days, with perioperative complication rates of 14.7, 24.1, 5.1, and 3.2%, respectively (5–7, 9), suggesting potentially higher complication rates when sICAS intervention is performed within 2 weeks of an AIS. Other studies have also identified intervention within 18 days, particularly for basilar artery stenosis rich in perforators, as a risk factor for perforator stroke (13). Research on timing for sICAS-related EC-IC bypass intervention also suggested a higher perioperative stroke rate in the early intervention group (within 7 days) compared to the delayed group (after 7 days) (14). The increased risk associated with early intervention may related to insufficient time for collateral circulation development, plaque instability, and disrupted blood–brain barrier at the infarction site, potentially increasing the risk of various stroke types, especially in posterior circulation lesions (13, 14). The classic 14-day window of highest recurrent stroke risk after symptomatic ICAS as reported in the WASID cohort (4). Consequently, the 2022 China expert consensus on endovascular interventional treatment for sICAS recommends performing endovascular treatment more than 14 days after the ischemic event for AIS patients with ICAS, except in cases of progressive stroke.

However, the stroke recurrence rate within 7 days after AIS can be as high as 11.5% (15). AIS accompanied by severe ICAS or intracranial atherosclerotic occlusion carries a 46–65% risk of early neurological deterioration (END) (16, 17). The only existing study focusing on surgical timing for minor stroke caused by ICAS reported no significant difference in perioperative complications between intervention within 14 days and after 14 days from the last AIS (18). Given this ongoing equipoise regarding the safety of early intervention, the recently published ANGEL-REBOOT trial further explored the role of emergent angioplasty or stenting following thrombectomy for acute large vessel occlusion (10). However, these studies did not include patients with multiple parallel severe stenoses or severe global brain injury, limiting their generalizability to real-world clinical scenarios. They also did not analyze the role of high-resolution MR, widely used clinically, in assessing culprit plaque stability. Therefore, determining the optimal timing for sICAS intervention in complex real-world situations remains an urgent issue in neurointervention.

## 2 Methods

### 2.1 Patients

A total of 211 consecutive patients with symptomatic intracranial atherosclerotic stenosis (sICAS) who underwent Endovascular treatment at the Second Affiliated Hospital of Xi’an Jiaotong University from January 1, 2019 to August 30, 2023 were retrospectively enrolled. The inclusion criteria were as follows: ① Age > 18 years; ② Digital subtraction angiography (DSA) revealed severe stenosis [70–99% stenosis degree, according to the WASID method (4)] in large intracranial arteries, including the intracranial segment of the internal carotid artery, M1 segment of the middle cerebral artery, basilar artery, and V4 segment of the vertebral artery; ③ Cerebral infarction in the blood supply area of the responsible artery occurred despite intensive medical treatment; ④ The ASITN/SIR score of collateral circulation under DSA was 2–3 (19); ⑤ Patients who underwent endovascular treatment on the target vessel. The exclusion criteria were as follows: ① Intracranial vascular stenosis caused by non-ICAS lesions, such as atrial fibrillation, vasculitis, moyamoya disease, and arterial dissection; ② Patients with intraoperative/postoperative immediate complications due to improper surgical operation; ③ Patients who underwent intervention more than 6 months after the last ischemic stroke episode; ④ Patients with incomplete clinical data or lost to follow-up. According to the interval from symptom onset to intervention, patients were assigned into early (<14 days) and delayed (>14 days) intervention groups. Since this is a retrospective study analyzing anonymized data, the institutional review board waived the requirement for informed consent.

### 2.2 Data collection

Demographic information, atherosclerotic risk factors, clinical presentation, laboratory results (including blood routine, coagulation profile, lipid panel, fasting blood glucose, glycosylated hemoglobin, liver and kidney function, uric acid, and homocysteine; the fibrinogen/albumin ratio [FAR] was calculated), imaging data, perioperative related data, and high-resolution vessel wall magnetic resonance imaging (HRVW MRI) data of the patients were collected from medical records by one neurologist and one neurointerventionalist.

### 2.3 Stenting procedure

All procedures were performed under general anesthesia. Anesthetics were chosen individually by the anesthesiologist and neurointerventionalist based on patient characteristics. Balloons and stents (Codman Enterprise, USA, or MicroPort Apollo, China) were selected according to lesion location, degree, and length of stenosis. Balloon diameter was chosen at 80% of the normal vessel diameter proximal to the lesion. Balloon inflation and deflation were performed slowly, with the entire process lasting over 1 min. After stent deployment or balloon angioplasty alone, the result was observed on the table for at least 10 min. Procedure success was defined as residual stenosis ≤30% and achievement of mTICI ≥2b flow.

### 2.4 Perioperative medication management

Preoperative: For patients with progressive stroke undergoing intervention within 72 h of onset, dual antiplatelet therapy (DAPT) loading doses were given. Other patients received DAPT guided by CYP2C19 genotype testing for 3–5 days preoperatively. CYP2C19 rapid metabolizers received clopidogrel (75 mg qd) plus aspirin (100 mg qd), whereas intermediate or poor metabolizers received ticagrelor (90 mg bid) or cilostazol (100 mg bid) plus aspirin (100 mg qd). Preoperative systolic blood pressure was controlled below 180 mmHg, fasting blood glucose below 10 mmol/L, and cardiopulmonary function was assessed suitable for general anesthesia.

Postoperative: Upon awakening from anesthesia, all patients continued DAPT based on CYP2C19 genotype for 3 months, followed by long-term single antiplatelet therapy. Blood pressure was maintained at 80–85% of the average preoperative blood pressure (over 3 days) for the first 48 h postoperatively. For patients without multiple parallel severe intracranial stenoses, long-term blood pressure was controlled within the normal range; otherwise, a tolerated target was set based on the overall cerebrovascular status. LDL was targeted at approximately 1.8 mmol/L. Patients were advised to quit smoking, limit alcohol, and reduce sedentary behavior.

### 2.5 Indications and timing for endovascular intervention

For choosing balloon angioplasty alone: short-segment stenosis (<10 mm), concentric non-calcified plaque on HR-MRI, absence of significant vessel tortuosity, and operator experience favoring simple dilation. For choosing stenting: suboptimal angioplasty result (residual stenosis >30%, flow-limiting dissection, or elastic recoil), or presence of high-risk plaque features (eccentric/irregular plaque, involvement of a major side branch). For timing of intervention: Except for progressive stroke, clinical stability (no new ischemic events for ≥72 h), adequate dual antiplatelet loading (3–5 days or a loading dose on the day prior), and absence of large infarct core (ASPECTS/pcASPECTS <6) or hemorrhagic transformation, early surgical intervention should be prioritized.

### 2.6 Follow-up

The first follow-up at the cerebrovascular disease specialty clinic occurred at 30 days (±3 days), including blood tests and TCD assessment of stented vessel flow. The second follow-up was at 90 days (±7 days), recording routine blood tests and the 90-day Modified Rankin Scale (mRS) score. The mRS score was dichotomized into favorable (mRS ≤ 2) or unfavorable (mRS > 2) outcomes.

### 2.7 Outcome indicators

The primary outcomes were any stroke or death within 30 days post-procedure and the 90-day unfavorable clinical outcome (mRS > 2) between the early and delayed intervention groups. Any stroke included ischemic stroke, asymptomatic intracranial hemorrhage (ICH), and symptomatic ICH (sICH). Ischemic stroke was defined as a new focal, sudden neurological deficit caused by cerebral infarction, confirmed by CT or MRI (9). Asymptomatic ICH referred to hemorrhage detected on follow-up CT without corresponding clinical symptoms. sICH was defined as subarachnoid, parenchymal, or intraventricular hemorrhage on MRI/CT associated with new neurological symptoms (altered consciousness, headache, focal signs) lasting >24 h or seizure (9). The secondary outcome was factors influencing the timing of intervention.

### 2.8 Statistical analysis

Data were analyzed using SPSS 26.0. Continuous variables are presented as mean ± standard deviation or median (IQR) based on distribution normality. Categorical variables are summarized as frequencies (percentages). Independent samples t-test, Mann–Whitney *U* test, Chi-square test, or Fisher’s exact test were used for comparisons as appropriate. Variables with *p* < 0.05 in univariate analysis were entered into multivariate logistic regression analysis using the “Forward: Conditional” method to assess their relationship with surgical timing selection. For the raw data with baseline imbalances, we employed multivariable regression analysis to explore factors influencing the baseline balance, and 1:2 subsequently performed propensity score matching to adjust for these differences. Adjust *p*-values using the Bonferroni method for multiple comparisons. A *p*-value < 0.05 was considered statistically significant.

## 3 Results

### 3.1 Baseline characteristics

**Figure 1:**
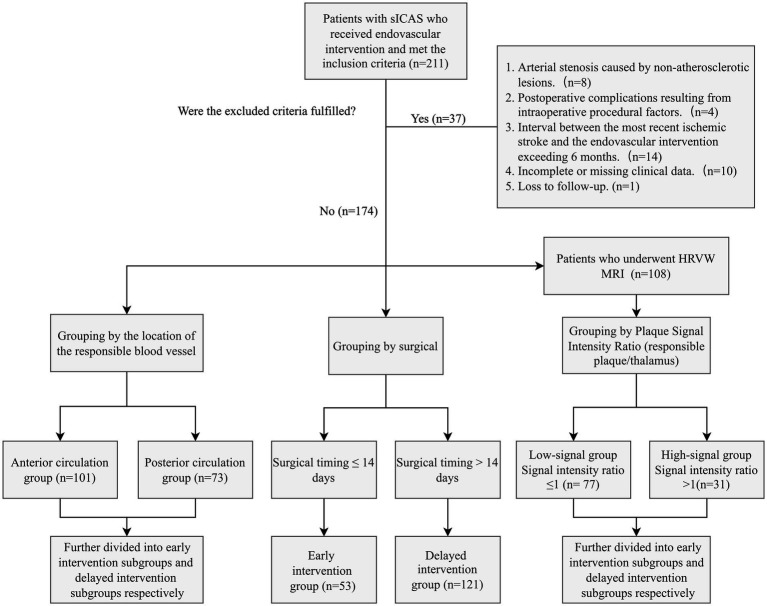
Patient selection and analysis flowchart.

A total of 211 sICAS patients met the inclusion criteria. After excluding 37 patients, 174 were included in the final analysis, including 8 patients with progressive stroke who underwent intervention within 72 h of onset. Stenting was performed in 169 patients, and balloon angioplasty alone in 5 patients. HRVW MRI was performed within 1 week before intervention in 108 patients. The patient selection and analysis flowchart is shown in Figure 1.

### 3.2 Clinical baseline characteristics by surgical timing

Baseline characteristics are shown in Table 1. Among the 174 patients, early intervention group 53 (30.5%) and delayed intervention group 121 (69.5%), 127 (73.0%) were male, with a mean age of 61.4 ± 8.5 years. Excluding the 8 patients with progressive stroke, CYP2C19 genotyping revealed 57.8% (96/166) were rapid metabolizers, 18.7% (31/166) were intermediate metabolizers, and 23.5% (39/166) were poor metabolizers. The clopidogrel resistance rate was 42.2% (70/166). Patients were divided into an Significant differences were found in admission NIHSS score and ASPECTS/pcASPECTS score between the two groups (*p* < 0.05). The early intervention group had higher preoperative NIHSS scores (*p* < 0.001) and lower ASPECTS/pcASPECTS scores (*p* = 0.023). Likely influenced by the preoperative NIHSS, the 24-h postoperative NIHSS score was also higher in the early intervention group (*p* < 0.001), although the median values were unchanged from preoperative levels. No significant differences were observed in other listed baseline characteristics (*p* > 0.05).

**Table 1:** Baseline clinical characteristics of early vs. delayed intervention groups.

Characteristic	Early intervention group (*n* = 53)	Delayed intervention group (*n* = 121)	*p*-value
Age (years), mean ± SD	62.6 ± 7.9	60.8 ± 8.7	0.199
Male, *n* (%)	35 (66.0)	92 (76.0)	0.172
BMI (kg/m^2^), mean ± SD	24.7 ± 2.5	24.9 ± 2.7	0.676
Smoking, *n* (%)	22 (41.5)	52 (43.0)	0.857
Alcohol consumption, *n* (%)	7 (13.2)	12 (9.9)	0.522
Past medical history, *n* (%)			
Coronary heart disease	6 (11.3)	21 (17.4)	0.312
Hypertension	39 (73.6)	93 (76.9)	0.642
Diabetes mellitus	22 (41.5)	51 (42.1)	0.937
Hyperlipidemia	28 (52.8)	53 (43.8)	0.272
Hyperhomocysteinemia	24 (45.3)	67 (55.4)	0.220
Previous stroke	12 (22.6)	28 (23.1)	0.943
Myocardial infarction	1 (1.9)	5 (4.1)	0.669
Hepatic/renal insufficiency	1 (1.9)	7 (5.8)	0.438
Preoperative condition			
NIHSS score, mean (IQR)	3.0 (1.0–5.0)	1.0 (0.0–2.5)	<0.001
ASPECTS/pcASPECTS score, mean (IQR)	8.0 (7.0–9.0)	9.0 (8.0–10.0)	0.023
Large-area cerebral infarction,ᵃ *n* (%)	3 (5.7)	4 (3.3)	0.437
Severe global brain damage before onset,ᵇ *n* (%)	11 (20.8)	41 (33.9)	0.082
Severe stenosis of multiple parallel vessels, *n* (%)	19 (35.8)	29 (24.0)	0.107
Clopidogrel resistance, *n* (%)	23 (46.0)	47 (40.5)	0.512
Local lesion characteristics			
Preoperative stenosis degree (%), mean (IQR)	99.0 (80.0–99.0)	90.0 (75.0–99.0)	0.147
Lesion length (mm), mean (IQR)	7.92 (7.23–9.11)	8.31 (6.96–9.53)	0.534
HR-MRI signal intensity (plaque/thalamus),ᶜ mean (IQR)	0.98 ± 0.05	0.89 ± 0.02	0.064
Lesion location, *n* (%)			
ICA	5 (9.4)	26 (21.5)	0.056
MCA	24 (45.3)	46 (38.0)	0.368
V4	13 (24.5)	28 (23.1)	0.843
BA	11 (20.8)	21 (17.4)	0.594
Intervention-related indicators			
Operation duration (minutes), mean (IQR)	90.0 (75.0–115.0)	95.0 (75.0–122.5)	0.314
NIHSS score within 24 h postoperatively, mean (IQR)	3.0 (1.0–6.0)	1.0 (0.0–3.0)	<0.001

Continuous variables are presented as median and IQR or mean and SD. Categorical variables are presented as frequencies (n) and percentages (%).

^a^Large-area cerebral infarction was defined as an ASPECTS/pcASPECTS score <6; ^b^Included patients with severe cerebral atrophy (defined as severe cerebral atrophy on visual assessment) and/or severe white matter lesions [defined as grade 3–4 on the van Swieten scale (32)]; ^c^HR-MRI signal intensity (plaque/thalamus) data were missing in 66 patients (37.9%), including 25 patients (47.2%) in the early intervention group and 41 patients (33.9%) in the delayed intervention group.

ASPECTS, Alberta stroke program early CT score; BA, Basilar artery; BMI, body mass index; HR-MRI, High-resolution magnetic resonance imaging; ICA, Internal carotid artery; MCA, Middle cerebral artery; NIHSS, National Institutes of Health Stroke Scale; V4, Vertebral artery V4 segment.

### 3.3 Auxiliary examination results by surgical timing

Laboratory results and echocardiographic EF values are shown in Supplementary Table 1. The early intervention group had significantly higher albumin-to-globulin ratio (1.6 vs. 1.5, *p* = 0.023), fasting blood glucose (5.5 vs. 5.2 mmol/L, *p* = 0.033), total cholesterol (4.0 vs. 3.5 mmol/L, *p* = 0.011), and LDL level (2.7 vs. 2.1 mmol/L, *p* = 0.003) compared to the delayed intervention group. Platelet count (193.0 vs. 207.0 × 10^9/L, *p* = 0.024) and APTT (22.6 vs. 23.7 s, *p* = 0.005) were lower in the early intervention group. No significant differences were found for other indicators (*p* > 0.05).

### 3.4 Multivariate analysis of factors influencing doctors’ choice of surgical timing and propensity score matching

Given the baseline imbalances but comparable perioperative outcomes, we investigated how the imbalanced baseline factors influenced the doctors’ choice of surgical timing. Factors with *p* < 0.05 in univariate analysis (Table 1 and Supplementary Table 1) and clinically relevant factors were entered into a multivariate model using the “Forward (Conditional)” method. After adjusting for confounders (including ASPECTS/pcASPECTS score, platelet count, fasting glucose, pre-existing severe global brain injury, culprit plaque high signal, preoperative stenosis degree), preoperative NIHSS score (OR = 0.819, 95% CI 0.685–0.978, *p* = 0.027), albumin-to-globulin ratio (A/G, OR = 0.149, 95% CI 0.024–0.936, *p* = 0.042), and LDL (OR = 0.394, 95% CI 0.211–0.737, *p* = 0.004) might be independent factors influencing the doctors’ decision on the timing of the intervention (Table 2). Receiver operating characteristic (ROC) curve analysis for these three timing-related variables suggested that NIHSS score <2.5, approximately equal to 3 (AUC = 0.664, 95% CI 0.570–0.757, *p* = 0.001), A/G < 1.53 (AUC = 0.603, 95% CI 0.509–0.697, *p* = 0.030), and LDL < 2.85 mmol/L (AUC = 0.642, 95% CI 0.551–0.733, *p* = 0.003) might influence the doctors’ decision for early intervention (Supplementary Figure 1 and Supplementary Table 2).

**Table 2:** Multivariate logistic regression analysis of factors influencing surgical timing.

Variable	OR value	95% CI	*p*-value
Model 1			
LDL	0.409	0.226–0.739	0.003
Model 2			
LDL	0.389	0.213–0.713	0.002
Preoperative NIHSS score	0.837	0.707–0.991	0.039
Model 3			
LDL	0.394	0.211–0.737	0.004
Preoperative NIHSS score	0.819	0.685–0.978	0.027
A/G	0.149	0.024–0.936	0.042

Confounding factors included ASPECTS/pcASPECTS score, platelet count, fasting blood glucose, severe global brain damage before onset, high-signal responsible plaques, and preoperative stenosis degree.

A/G, albumin-globulin ratio; LDL, Low-density lipoprotein; OR, odds ratios; 95% CI, 95% confidence intervals.

Due to baseline imbalance, multivariable analysis revealed that preoperative NIHSS score, LDL, and albumin-globulin ratio remained statistically different (Table 2). We therefore employed propensity score matching to adjust baseline characteristics (Table 3 and Supplementary Table 1).

**Table 3:** Baseline clinical characteristics of early vs. delayed intervention groups (after matching).

Characteristic	After matching		
	Early intervention group (*n* = 42)	Delayed intervention group (*n* = 69)	*p*-value
Age (years), mean ± SD	63.2 ± 7.3	61.0 ± 8.1	0.154
Male, *n* (%)	26 (61.9)	52 (75.4)	0.132
BMI (kg/m^2^), mean ± SD	25.1 ± 2.6	24,8 ± 2.8	0.578
Smoking, *n* (%)	17 (40.5)	29 (42.0)	0.872
Alcohol consumption, *n* (%)	6 (14.3)	4 (5.8)	0.130
Past medical history, *n* (%)			
Coronary heart disease	3 (7.1)	12 (17.4)	0.126
Hypertension	30 (71.4)	54 (78.3)	0.416
Diabetes mellitus	17 (40.5)	30 (43.5)	0.756
Hyperlipidemia	19 (45.3)	33 (47.8)	0.791
Hyperhomocysteinemia	17 (40.5)	37 (53.6)	0.179
Previous stroke	10 (23.8)	21 (30.4)	0.451
Myocardial infarction	0 (0.0)	1 (1.4)	1.000
Hepatic/renal insufficiency	0 (0.0)	2 (2.9)	0.525
Preoperative condition			
NIHSS score, mean (IQR)	2 (0–4)	2 (0–3.5)	0.469
ASPECTS/pcASPECTS score, mean (IQR)	8 (7.7–9.3)	9 (7–10)	0.119
Large-area cerebral infarction,ᵃ *n* (%)	3 (7.1)	2 (2.9)	0.296
Severe global brain damage before onset,ᵇ *n* (%)	9 (21.4)	26 (37.7)	0.074
Severe stenosis of multiple parallel vessels, *n* (%)	16 (38.1)	18 (26.1)	0.183
Clopidogrel resistance, *n* (%)	19 (47.5)	27 (42.4)	0.596
Local lesion characteristics			
Preoperative stenosis degree (%), mean (IQR)	90.0 (80.0–99.0)	90.0 (77.4–99.0)	0.665
Lesion length (mm), mean (IQR)	8.01 (7.24–9.42)	8.18 (6.54–9.45)	0.627
HR-MRI signal intensity (plaque/thalamus),ᶜ mean (IQR)	0.99 ± 0.29	0.89 ± 0.19	0.254
Lesion location, *n* (%)			
ICA	4 (9.5)	16 (23.2)	0.069
MCA	17 (40.5)	25 (36.2)	0.655
V4	12 (28.6)	17 (24.6)	0.647
BA	9 (21.4)	11 (15.9)	0.466
Intervention-related indicators			
Operation duration (minutes), mean (IQR)	86.0 (73.8–107.5)	100.0 (75.0–120.0)	0.109
NIHSS score within 24 h postoperatively, mean (IQR)	2.0 (0–4.3)	1.0 (0–3.0)	0.252

Continuous variables are presented as median and IQR or mean and SD. Categorical variables are presented as frequencies (n) and percentages (%).

^a^Large-area cerebral infarction was defined as an ASPECTS/pcASPECTS score <6; ^b^Included patients with severe cerebral atrophy (defined as severe cerebral atrophy on visual assessment) and/or severe white matter lesions [defined as grade 3–4 on the van Swieten scale (32)]; ^c^HR-MRI signal intensity (plaque/thalamus) data were missing in 66 patients (37.9%), including 25 patients (47.2%) in the early intervention group and 41 patients (33.9%) in the delayed intervention group.

ASPECTS, Alberta stroke program early CT score; BA, Basilar artery; BMI, body mass index; HR-MRI, High-resolution magnetic resonance imaging; ICA, Internal carotid artery; MCA, Middle cerebral artery; NIHSS, National Institutes of Health Stroke Scale; V4, Vertebral artery V4 segment.

### 3.5 Perioperative stroke, death, and 90-day outcomes by surgical timing

Among the 174 patients, the overall incidence of any stroke or death within 30 days was 9.2% (16/174), with no deaths. Six events occurred in the early intervention group (37.5% of events), and nine patients had unfavorable 90-day outcomes (56.3% of event patients). No significant differences were found in clinical outcomes between the two groups (all *p* > 0.05). After applying propensity score matching to adjust for baseline characteristics (Table 3 and Supplementary Table 1), we found that there remained no significant difference in primary outcomes between the early intervention group and the late intervention group (Table 4). Additionally, we will analyze the relationship between surgical time as a continuous variable and the main clinical outcomes (Supplementary Table 3), and the results show that there is no significant nonlinear trend in surgical risk over time. We also conducted sensitivity analyses with 7 and 21 days as the boundary points, and the results showed that there were no significant differences in primary clinical outcomes (any stroke/death within 30 days or 90-day unfavorable outcome) among various grouping methods (all *p* > 0.05) (Supplementary Table 4).

**Table 4:** Comparison of perioperative stroke/death and clinical outcomes by surgical timing.

Outcome indicator	Before matching			After matching		
	Early intervention group (*n* = 53)	Delayed intervention group (*n* = 121)	*p*-value	Early intervention group (*n* = 42)	Delayed intervention group (*n* = 69)	*p*-value
Any stroke or death, *n* (%)	6 (11.3)	10 (8.3)	0.521	4 (9.5)	6 (8.7)	0.883
Symptomatic postoperative stroke-related complications, *n* (%)	5 (9.4)	9 (7.4)	0.763	3 (7.1)	6 (8.7)	0.771
Ischemic stroke, *n* (%)	5 (9.4)	6 (5.0)	0.313	3 (7.1)	3 (4.3)	0.671
Symptomatic intracranial hemorrhage (sICH), *n* (%)	0 (0.0)	3 (2.5)	0.554	0 (0.0)	3 (4.3)	0.288
Asymptomatic intracranial hemorrhage, *n* (%)	1 (1.9)	1 (0.8)	0.518	1 (2.4)	0 (0.0)	0.378
Unfavorable outcome at 90 days, *n* (%)	10 (18.9)	13 (10.7)	0.145	8 (19.0)	11 (15.9)	0.674

Among the 16 stroke patients, 11 (68.8%) had symptomatic new infarctions. Notably, all 11 were due to perforator occlusion or in-stent thrombosis: 6 in the mid-basilar artery, 1 in M1 perforators post-stenting. Intervention timing for these were 14, 13, 6, 69, 15, 14, and 42 days; symptom onset post-procedure were 36 h, immediate, 24 h, 1 h, immediate and 36 h. The patient with symptoms at 36 h, despite tetraplegia, recovered completely by 3 months. One patient with dysphagia had a high-signal culprit plaque on preoperative HR-MRI (intervention day 13). Others presented with ataxia (2), asymmetric limb weakness (2), or mild hemiparesis (1). Four ischemic events were in-stent thromboses (intervention days 0, 133, 67, 19; symptom onset 10d, 20d, 15d, 3d post-op), all confirmed by DSA and recanalized after short-term IV antiplatelet therapy. Any ICH occurred in 5 patients (31.3% of events): 2 asymptomatic ICHs (convexal SAH on 24 h CT, intervention days 0, 22) and 3 sICHs (intervention days 22, 15, 52; symptom onset 4 h, 2 h, 1.5 h post-op), presenting with slurred speech, contralateral weakness; one had seizures. Two of the three sICH patients had severe pre-existing global brain injury.

### 3.6 Influence of culprit vessel HRVW MRI plaque signal intensity on surgical timing

Among 108 patients who underwent HRVW MRI within 7 days preoperatively, 31 with a plaque-to-thalamus signal intensity ratio >1 was defined as the high-signal group, and 77 with a ratio ≤1 as the low-signal group. In the high-signal group (early intervention *n* = 10, delayed *n* = 21), only one patient in the early intervention group had a symptomatic new infarction (1/10 vs. 0, *p* = 0.323); 90-day unfavorable outcomes were 1/10 (10.0%) vs. 1/21 (4.8%), *p* = 1.000. In the low-signal group (early *n* = 18, delayed *n* = 59), any stroke/death rates were 3/18 (16.7%) vs. 7/59 (11.9%), *p* = 0.596; 90-day unfavorable outcomes were 3/18 (16.7%) vs. 7/59 (11.9%), *p* = 0.596. No significant differences in clinical outcomes were found within different signal intensity groups stratified by timing (all *p* > 0.05) (Supplementary Tables 5, 6).

### 3.7 Influence of culprit vessel location on surgical timing

Patients were categorized into anterior circulation (*n* = 101) and posterior circulation (*n* = 73) groups. Any stroke/death in the anterior circulation was 10 cases (9.9%): 2 (6.9%) in early vs. 8 (11.1%) in delayed intervention, *p* = 0.521. In the posterior circulation, any stroke/death was 6 cases (8.2%): 4 (16.7%) in early vs. 2 (4.1%) in delayed intervention, *p* = 0.086. The posterior circulation early intervention group had a significantly higher rate of unfavorable outcomes compared to the delayed intervention group (29.2% vs. 8.2%, *p* = 0.033). No other outcome measures differed significantly between timing groups within anterior or posterior circulation (*p* > 0.05) (Supplementary Tables 7, 8). Among the 7 posterior circulation early-intervention patients with unfavorable 90-day outcomes, 6 (85.7%) had pre-existing severe baseline deficits (baseline mRS 3–5), and 3 (42.8%) developed new perforator-related infarcts confirmed by diffusion-weighted MRI. No cases of in-stent thrombosis or symptomatic hemorrhage occurred in this subgroup.

## 4 Discussion

Determining the optimal timing for intervention in sICAS patients remains a critical clinical challenge. This study aimed to identify clinical factors associated with doctors’ decisions on surgical timing by retrospectively comparing the characteristics and outcomes of patients who underwent early versus delayed intervention. The overall perioperative 30-day stroke/death rate was 9.2% (0% mortality). Using a 14-day threshold, the rates of any stroke/death within 30 days (11.3% vs. 8.3%) and unfavorable outcomes at 90 days (18.9% vs. 10.7%) did not differ significantly between early and delayed intervention groups (*p* > 0.05). Preoperative NIHSS score, serum albumin-to-globulin ratio, and LDL level might be factors significantly attracted the doctors’ decision on the timing of the intervention (OR = 0.819, 0.149, 0.394). NIHSS score ≤3, albumin-to-globulin ratio <1.53, and LDL < 2.85 mmol/L may be predictive thresholds for selecting early intervention in our cohort. For posterior circulation lesions, early intervention was associated with significantly worse outcomes compared to delayed intervention (29.2% vs. 8.2%, *p* = 0.033). Notably, multiple parallel severe stenoses, severe pre-existing global brain injury, and high-signal plaques on HR-MRI did not increase perioperative stroke risk or influence timing selection in this cohort. Potential explanations include: effective antiplatelet therapy, mechanical plaque modification, and individualized perioperative blood pressure management may offset the periprocedural risk of these high-risk features. Additionally, clinicians may have preferentially assigned high-risk patients to delayed intervention based on clinical experience, introducing selection bias. Furthermore, the limited sample size and low event rate in subgroup analyses restricted statistical power, the generalizability of this conclusion requires cautious interpretation and necessitates validation by future research with larger cohorts.

The increased risk associated with early intervention appears linked to higher preoperative NIHSS scores, which often correlate with larger infarct volume in anterior circulation strokes. These patients may have concomitant cerebral edema in the early AIS phase, and the infarct zone may contain fragile neovascularure and a disrupted blood–brain barrier, increasing the risk of hemorrhagic transformation post-intervention, with sICH being a critical complication affecting prognosis. However, our study population primarily consisted of minor strokes (median NIHSS ≤3) with small infarct volumes (median ASPECTS/pcASPECTS 8–9). In such patients, not only were favorable outcome rates similar between early and delayed intervention, but the incidence of sICH was also very low (2.5%), consistent with studies using ASPECTS to predict hemorrhage risk after reperfusion, which reported a 2.6% sICH rate for ASPECTS 8–10 (20). These findings suggest that for minor stroke patients with small infarct volumes, endovascular treatment may not be strictly time-limited, and early intervention could be considered a viable approach.

Previous literature indicates that global brain health significantly impacts overall prognosis in AIS patients (21). Therefore, we included global brain injury in our analysis. Our study included 29.9% (52/174) of patients with severe pre-existing global brain injury (severe atrophy and/or severe white matter lesions). The perioperative stroke rate did not differ between timing groups for these patients, although their proportion was higher in the delayed intervention group (33.9% vs. 20.8%), suggesting that delayed intervention might be preferable to achieve similar safety outcomes in this subgroup. Furthermore, based on our clinical experience, meticulous perioperative management is crucial for achieving favorable clinical outcomes in these patients.

Additionally, the increased risk associated with early intervention was linked to higher LDL and albumin-to-globulin ratio. High LDL is a well-established stroke risk factor, promoting atherosclerosis, plaque rupture, thrombosis, and vascular dysfunction, leading to ischemic strokes. For patients with elevated preoperative LDL, delayed intervention after intensive statin therapy might be preferable.

Due to anatomical differences, the posterior circulation has a less complete blood–brain barrier (22, 23) and relatively incomplete sympathetic innervation of the vertebrobasilar system, leading to weaker autoregulation in small cerebral arteries and reduced protection of the BBB in corresponding areas. This makes the posterior circulation territory susceptible to various pathologies, affecting clinical outcomes (24, 25). Our study also found that early intervention in the posterior circulation was associated with worse outcomes, potentially related to the near-significant difference in any stroke/death between early and delayed intervention groups for posterior circulation (16.7% vs. 4.1%, *p* = 0.086). Given the small sample size (*n* = 24) and observational nature, this finding is only hypothesis-generating: posterior circulation lesions may be more vulnerable to early intervention, requiring validation in larger studies. In addition, the posterior circulation BA is rich in perforator, perforator occlusion is a major cause of complications in sICAS endovascular treatment; in the SAMMPRIS trial, 12 of 19 ischemic stroke complications (63.2%) were perforator occlusions (5). The CASSISS trial excluded patients with pre-operative perforator stroke (7). A study of 174 BA stenting patients found a higher proportion of plaque enhancement in proximal BA stenosis, particularly at the vertebrobasilar junction, which also had the highest proportion of perforator strokes; these plaques were often distributed on the lateral wall of the BA and were enhanced (26). Slow balloon inflation/deflation (27), perioperative intravenous tirofiban (28), and submaximal angioplasty are proposed methods to reduce such complications (29). However, the optimal dose and regimen for perioperative tirofiban remain unclear. Some patients in our study received intermittent intracatheter boluses of low-dose tirofiban or intravenous infusion, yet perforator occlusion still accounted for 63.6% (7/11) of new ischemic strokes. Preoperative assessment of plaque burden and remodeling pattern in perforator-rich MCA using HR-MRI has been suggested to potentially reduce perforator stroke (30). Theoretically, if pre-operative imaging could clearly determine that perforators originate opposite the plaque, the risk of occluding them during intervention might be lower, but this may require higher-field MRI scanners (e.g., >5T) than the commonly used 3 T machines.

Previous studies suggest that high signal intensity on HR-MRI, intraplaque hemorrhage, and plaque enhancement are markers of plaque instability (31). Our analysis of different plaque signals found no significant impact on perioperative stroke complications or surgical timing choice. In the 31 high-signal patients, 1/10 (10.0%) in the early intervention group had an ischemic stroke; in the low-signal group, 8/77 (10.4%) had ischemic strokes (3/18 [16.7%] early vs. 5/59 [8.5%] delayed), suggesting cautious interpretation of high signal as solely indicating unstable plaque in this context. Potential reasons for this finding include: ① The small sample size of the high-signal group and even smaller number of complication events; ② Limited information from the 3T HR-MRI used, with relatively thick slices (0.4 mm); and ③ The influence of blood flow velocity on vessel wall signal intensity.

Despite the novel findings of the present study, several limitations should be acknowledged. First, the single-center retrospective design and predominantly East-Asian cohort limit generalizability. Second, clinicians might have deliberately delayed intervention for sicker patients, although we performed multivariable adjustment to mitigate this, residual confounding cannot be entirely ruled out. Third, inclusion of a few progressive-stroke patients may introduce bias. Fourth, approximately 40% of patients lacked HR-VWI data, may weaken plaque-signal analyses. Fifth, the 14-day cutoff, though consensus-based, remains somewhat arbitrary. Sixth, the subgroup analysis in posterior circulation lesions had limited statistical power due to the small number of events (*n* = 24 in the early group), these findings should be considered exploratory and require validation in larger, prospective cohorts. Finally, the follow-up period was limited to 90 days, and the lack of long-term functional outcomes and restenosis data warrants further investigation in future studies.

## 5 Conclusion

In this retrospective, real-world, exploratory study, we observed that for anterior circulation sICAS patients with minor stroke (NIHSS ≤3), high ASPECTS/pcASPECTS scores (8–9), and well-controlled LDL, the timing of endovascular treatment may not be restricted. For the posterior circulation, particularly BA lesions, delayed intervention might be safer. Multiple parallel severe stenoses, severe pre-existing global brain injury, and plaque high-signal intensity did not influence on the surgical timing. Due to the inherent limitations of the retrospective design and the limited number of outcome events in subgroup analyses, the conclusions of this study should be interpreted cautiously and require validation in future prospective studies.

## Data Availability

The raw data supporting the conclusions of this article will be made available by the authors, without undue reservation.
