# Supplementary material for: Optimal timing of endovascular treatment for symptomatic intracranial atherosclerotic stenosis: a real world single center study
Source: Front Neurol. 2026 Apr 22;17:1749046. doi: 10.3389/fneur.2026.1749046 (PMC13143744; doi:10.3389/fneur.2026.1749046)
Supplement: Supplementary file 1 [file Supplementary_file_1.DOCX]

**Optimal Timing of Endovascular Treatment for Symptomatic Intracranial Atherosclerotic Stenosis: A Real World Single Center Study**

**Supplemental Materials**

**TABLES**

**Online Supplemental Table 1** Key Auxiliary Examination Results of Early vs. Delayed intervention Groups

| **Indicator** | **Before Matching** | | | **After Matching** | | |
| --- | --- | --- | --- | --- | --- | --- |
|  | **Early intervention Group (n=53)** | **Delayed intervention Group (n=121)** | **p-Value** | **Early intervention Group (n=42)** | **Delayed intervention Group (n=69)** | **p-Value** |
| WBC (×10⁹/L), mean (IQR) | 6.7（5.3-8.1） | 6.5（5.5-7.7） | 0.943 | 6.7（5.4-7.9） | 6.4（5.4-7.5） | 0.750 |
| PLT (×10⁹/L), mean (IQR) | 193.0（148.5-210.0） | 207.0（162.5-256.5） | 0.024 | 193.5（148.0-216.0） | 207.0（170.0-253.0） | 0.370 |
| Hemoglobin (g/L), mean ± SD | 138.0±15.7 | 139.5±14.4 | 0.528 | 138.8±16.1 | 141.4±13.7 | 0.365 |
| Neutrophils (×10⁹/L), mean (IQR) | 4.5（3.2-5.7） | 4.3（3.3-5.2） | 0.827 | 4.2（3.3-5.6） | 4.2（3.3-5.1） | 0.789 |
| Monocytes (×10⁹/L), mean (IQR) | 0.4（0.3-0.5） | 0.4（0.3-0.6） | 0.229 | 0.4（0.3-0.5） | 0.4（0.3-0.5） | 0.480 |
| Lymphocytes (×10⁹/L), mean (IQR) | 1.6（1.2-1.9） | 1.6（1.3-2.1） | 0.169 | 1.5（1.2-1.9） | 1.5（1.3-2.0） | 0.627 |
| ALT (IU/L), mean (IQR) | 20.0（14.0-28.2） | 21.0（15.0-35.0） | 0.238 | 20.0（13.0-27.3） | 21.0（15.0-31.0） | 0.290 |
| AST (IU/L), mean (IQR) | 20.0（17.2-25.5） | 22.0（18.0-29.5） | 0.189 | 20.0（17.8-22.7） | （21.0（17.0-28.0）） | 0.404 |
| TP (g/L), mean (IQR) | 64.8（61.9-69.1） | 66.4（60.7-69.4） | 0.633 | 66.0（62.5-69.3） | 65.8（60.7-68.5） | 0.146 |
| Albumin (g/L), mean (IQR) | 39.6（38.0-42.4） | 39.5（36.9-42.1） | 0.371 | 39.8（38.2-43.0） | 39.8（36.8-41.9） | 0.154 |
| Globulin (g/L), mean (IQR) | 24.7（21.8-27.1） | 25.6（23.3-28.7） | 0.053 | 25.6（23.5-27.4） | 25.4（22.6-27.5） | 0.761 |
| A/G, mean ± SD | 1.6±0.3 | 1.5±0.3 | 0.023 | 1.6±0.3 | 1.6±0.3 | 0.568 |
| FPG (mmol/L), mean (IQR) | 5.5（4.8-6.9） | 5.2（4.5-6.3） | 0.033 | 5.4（4.7-6.4） | 5,2（4,5-6.2） | 0.242 |
| eGFR (mL/min/1.73m²), mean (IQR) | 98.9（91.5-111.9） | 104.2（96.0-112.0） | 0.211 | 98.2（91.2-109.5） | 103.9（95.9-113.5） | 0.059 |
| Uric acid (μmol/L), mean (IQR) | 275.6（234.0-351.5） | 289.0（233.5-344.5） | 0.557 | 276.6（243.0-351.3） | 288.0（232.5-342.5） | 0.862 |
| TC (mmol/L), mean (IQR) | 4.0（3.2-4.6） | 3.5（2.9-4.2） | 0.011 | 3.9（3.2-4.5） | 3.9（3.1-4.4） | 0.862 |
| TG (mmol/L), mean (IQR) | 1.3（1.1-1.5） | 1.3（1.0-1.7） | 0.871 | 1.3（1.1-1.5） | 1.4（1.1-2.0） | 0.061 |
| HDL (mmol/L), mean (IQR) | 1.0（0.9-1.1） | 1.0（0.8-1.2） | 0.751 | 1.0（0.9-1.2） | 1.0（0.8-1.2） | 0.664 |
| LDL (mmol/L), mean (IQR) | 2.7（1.9-3.0） | 2.1（1.7-2.7） | 0.003 | 2.5（1.9-3.0） | 2.4（1.9-2.9） | 0.549 |
| VLDL (mmol/L), mean (IQR) | 0.3（0.2-0.5） | 0.3（0.2-0.4） | 0.683 | ~~0.3（0.2-0.4）~~ | ~~0.4（0.3-0.5）~~ | ~~0.020~~ |
| HCY (μmol/L), mean (IQR) | 15.3（12.4-21.6） | 16.1（12.0-22.0） | 0.970 | 15.0（12.2-21.5） | 15.5（11.8-21.7） | 0.794 |
| HbA1c (%), mean (IQR) | 6.0（5.4-7.2） | 6.1（5.5-7.1） | 0.956 | 6.0（5.4-7.1） | 6.1（5.5-7.2） | 0.843 |
| D-dimer (ng/mL), mean (IQR) | 540.0（370.0-720.0） | 450.0（350.0-635.0） | 0.190 | 555.0（365.0-850.0） | 470.0（330.0-690.0） | 0.296 |
| Fibrinogen (mg/dL), mean (IQR) | 280.0（253.0-330.0） | 300.0（249.0-351.0） | 0.482 | 302.5（257.0-333.0） | 300.0（254.0-351.0） | 0.973 |
| FAR, mean (IQR) | 7.3（5.9-8.7） | 7.6（6.3-9.2） | 0.329 | 7.5（6.1-8.7） | 7.6（6.6-8.9） | 0.519 |
| EF(%), mean ± SD | 66.2±4.5 | 66.1±5.8 | 0.937 | 65.9±4.3 | 66.4±5.3 | 0.560 |

·A/G, albumin-globulin ratio; ALT, alanine aminotransferase; AST, aspartate aminotransferase; EF, ejection fraction; eGFR, estimated glomerular filtration rate; FAR, fibrinogen / albumin Ratio; FPG, fasting plasma glucose; HbA1c, glycated hemoglobin; HCY, homocysteine; HDL, high-density lipoprotein; LDL, low-density lipoprotein; PLT, platelets; TC, total cholesterol; TG, triglycerides; TP, total protein; VLDL, very low-density lipoprotein; WBC, white blood cells.

**Online Supplemental Table 2** Influenced Value of Variables for Selecting Early intervention

| **Variable** | **AUC (95% CI)** | **Cutoff Value** | **Sensitivity** | **Specificity** | **p-Value** |
| --- | --- | --- | --- | --- | --- |
| Preoperative NIHSS score | 0.664（0.570-0.757） | 2.50 | 0.59 | 0.75 | 0.001 |
| A/G | 0.603（0.509-0.697） | 1.53 | 0.72 | 0.51 | 0.030 |
| LDL | 0.642（0.551-0.733） | 2.85 | 0.45 | 0.82 | 0.003 |

·A/G, albumin-globulin ratio; AUC, Area Under the ROC Curve; CI, Confidence Interval; LDL, low-density lipoprotein; NIHSS, National Institutes of Health Stroke Scale; ROC, Receiver Operating Characteristic.

**Online Supplemental Table 3** Logistic Regression Analysis of Comparison of Perioperative Stroke/Death and Clinical Outcomes by Surgical Timing

| Variable | OR Value | 95% CI | p-Value |
| --- | --- | --- | --- |
| Any stroke or death, n (%) | 1.005 | 0.987-1.023 | 0.561 |
| Symptomatic postoperative stroke-related complications, n (%) | 1.008 | 0.991-1.027 | 0.359 |
| Ischemic stroke, n (%) | 1.010 | 0.990-1.029 | 0.332 |
| Symptomatic intracranial hemorrhage (sICH), n (%) | 1.002 | 0.961-1.045 | 0.922 |
| Asymptomatic intracranial hemorrhage, n (%) | 0.938 | 0.802-1.098 | 0.428 |
| Unfavorable outcome at 90 days, n (%) | 0.984 | 0.961-1.008 | 0.192 |

**Online Supplemental Table 4** Comparison of Perioperative Stroke/Death and Clinical Outcomes by Surgical Timing (7 days and 21 days cutoffs)

| **Outcome Indicator** | **7-days cutoff** | | | **21-days cutoff** | | |
| --- | --- | --- | --- | --- | --- | --- |
|  | **Early intervention Group (n=15)** | **Delayed intervention Group (n=159)** | **p-Value** | **Early intervention Group (n=99)** | **Delayed intervention Group (n=75)** | **p-Value** |
| Any stroke or death, n (%) | 3（20.0） | 13（8.2） | 0.130 | 9（9.1） | 7（9.3） | 0.956 |
| Symptomatic postoperative stroke-related complications, n (%) | 2（13.3） | 12（7.5） | 0.431 | 8（8.1） | 6（8.0） | 0.985 |
| Ischemic stroke, n (%) | 2（13.3） | 9（5.7） | 0.243 | 7（7.1） | 4（5.3） | 0.641 |
| Symptomatic intracranial hemorrhage (sICH), n (%) | 0（0.0） | 3（1.9） | 1.000 | 1（1.0） | 2（2.7） | 0.578 |
| Asymptomatic intracranial hemorrhage, n (%) | 1（6.7） | 1（0.6） | 0.165 | 1（1.0） | 1（1.3） | 1.000 |
| Unfavorable outcome at 90 days, n (%) | 3（20.0） | 20（12.6） | 0.417 | 16（16.2） | 7（9.3） | 0.188 |

**Online Supplemental Table 5** Comparison of Perioperative Complications and Clinical Outcomes Between Early intervention and Late intervention in the High-Signal Group

|  | **Early intervention group (n=10)** | **Late intervention group (n=21)** | **p-Value** |
| --- | --- | --- | --- |
| Postoperative stroke-related complications, n (%) | 1（10.0） | 0（0.0） | 0.323 |
| Symptomatic new cerebral infarction, n (%) | 1（10.0） | 0（0.0） | 0.323 |
| Symptomatic intracranial hemorrhage, n (%) | 0 | 0 | - |
| Unfavorable outcome at 90 days, n (%) | 1（10.0） | 1（4.8） | 1.000 |

**Online Supplemental Table 6** Comparison of Perioperative Complications and Clinical Outcomes Between Early intervention and Late intervention in the Low-Signal Group

|  | **Early intervention group (n=18)** | **Late intervention group (n=59)** | **p-Value** |
| --- | --- | --- | --- |
| Postoperative stroke-related complications, n (%) | 3（16.7） | 7（11.9） | 0.596 |
| Symptomatic new cerebral infarction, n (%) | 3（16.7） | 5（8.5） | 0.319 |
| Symptomatic intracranial hemorrhage, n (%) | 0（0.0） | 2（3.4） | 1.000 |
| Unfavorable outcome at 90 days, n (%) | 3（16.7） | 7（11.9） | 0.596 |

**Online Supplemental Table 7** Comparison of Perioperative Complications and Clinical Outcomes Between Early intervention and Late intervention in the Posterior Circulation Group

|  | **Early intervention group (n=24)** | **Late intervention group (n=49)** | **p-Value** |
| --- | --- | --- | --- |
| Postoperative stroke-related complications, n (%) | 4（16.7） | 2（4.1） | 0.086 |
| Symptomatic new cerebral infarction, n (%) | 4（16.7） | 2（4.1） | 0.086 |
| Symptomatic intracranial hemorrhage, n (%) | 0 | 0 | - |
| Unfavorable outcome at 90 days, n (%) | 7（29.2） | 4（8.2） | 0.033 |

**Online Supplemental Table 8** Comparison of Perioperative Complications and Clinical Outcomes Between Early intervention and Late intervention in the Anterior Circulation Group

|  | **Early intervention group (n=29)** | **Late intervention group (n=72)** | **p-Value** |
| --- | --- | --- | --- |
| Postoperative stroke-related complications, n (%) | 1（3.4） | 7（9.7） | 0.433 |
| Symptomatic new cerebral infarction, n (%) | 1（3.4） | 4（5.6） | 1.000 |
| Symptomatic intracranial hemorrhage, n (%) | 0（0.0） | 3（4.2） | 0.555 |
| Unfavorable outcome at 90 days, n (%) | 3（10.3） | 9（12.5） | 1.000 |

**FIGURE**


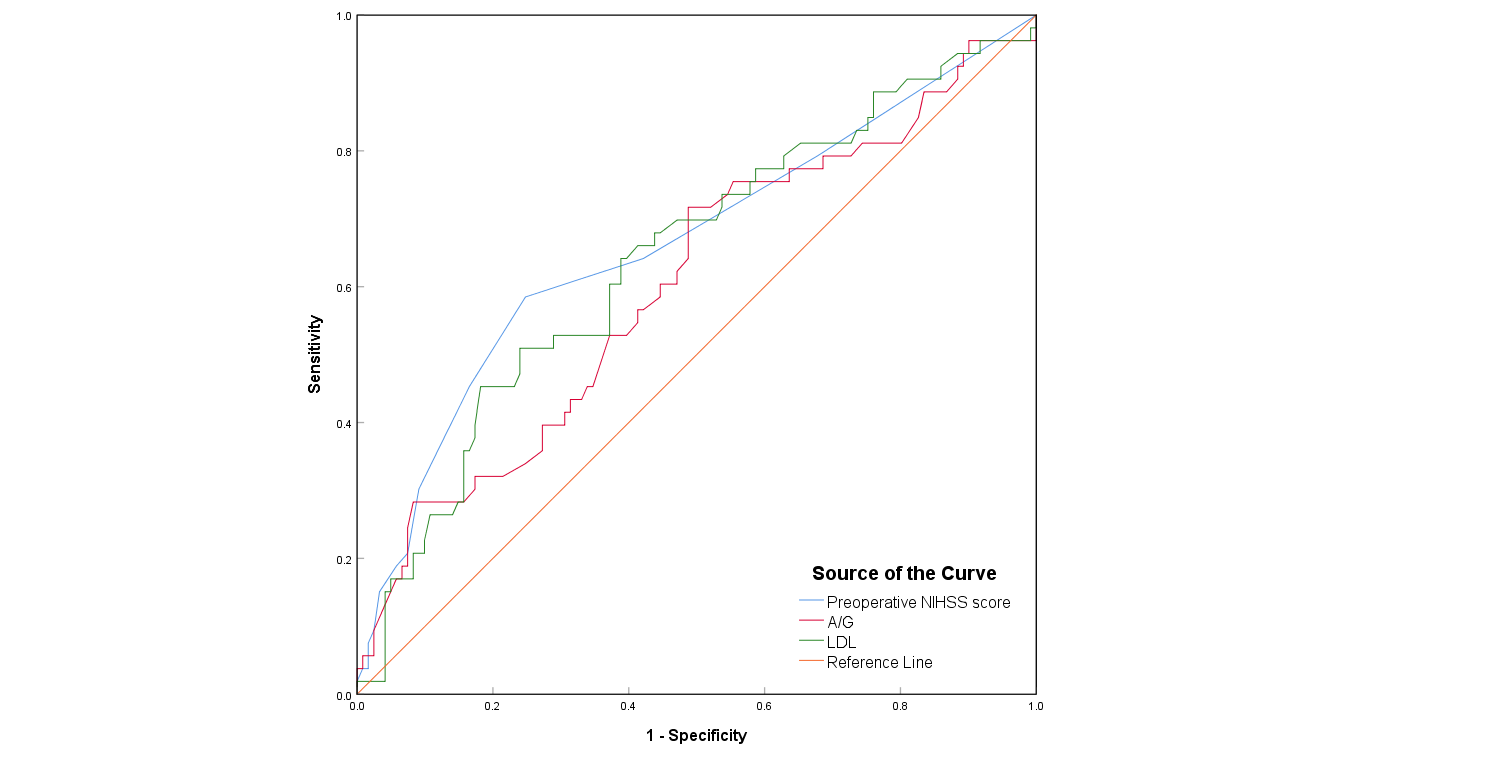


**Online Supplemental Figure 1** ROC Curves for Variables Related to Surgical Timing Selection.

·The ROC curves were generated for preoperative NIHSS score, albumin-globulin ratio, and LDL level. The diagonal line represents the reference line.

·A/G, albumin-globulin ratio; LDL, low-density lipoprotein; NIHSS, National Institutes of Health Stroke Scale.
